# Nucleoside diphosphate kinase strongly promotes GDP and ADP metabolism in the cell and affects endogenous proton leak in mitochondria – the kinase is hampered by oxidative phosphorylation inhibitors

**DOI:** 10.1080/14756366.2025.2520611

**Published:** 2025-07-17

**Authors:** Andrzej M. Woyda-Ploszczyca

**Affiliations:** Department of Bioenergetics, Institute of Molecular Biology and Biotechnology, Faculty of Biology, Adam Mickiewicz University, Poznan, Poland

**Keywords:** ADP/ATP carrier, nucleotide metabolism, mitochondria, nucleoside-diphosphate kinase (NDPK), proton (H^+^) leak

## Abstract

Rapid GDP metabolism in mitochondria isolated from wild-type yeast is postulated. The hallmark of exogenous GDP is convergence with the effect of exogenous ADP, typically inducing oxidative phosphorylation (OXPHOS). The GDP-provoked changes in the presence of ATP, i.e. increased respiratory rate accompanied by decreased inner mitochondrial membrane electrical potential, were curtailed by OXPHOS inhibitors, such as carboxyatractyloside, which apparently merged the GDP effect with OXPHOS. However, all performed tests indicated that the response of mitochondria to GDP is indirect and involves two steps. First, GDP is transphosphorylated *via* nucleoside diphosphate kinase (NDPK), ATP + GDP → ADP + GTP, which is followed by ADP-induced OXPHOS. Importantly, in mitochondria isolated from mutant yeast with a deleted NDPK gene, the stimulatory effect of GDP was eliminated. Therefore, a prerequisite for GDP metabolic action is the cooperation of NDPK with the OXPHOS apparatus. This biological model can help elucidate the molecular basis of some diseases treatment, such as cancer.

## GRAPHICAL ABSTRACT

**Figure:**
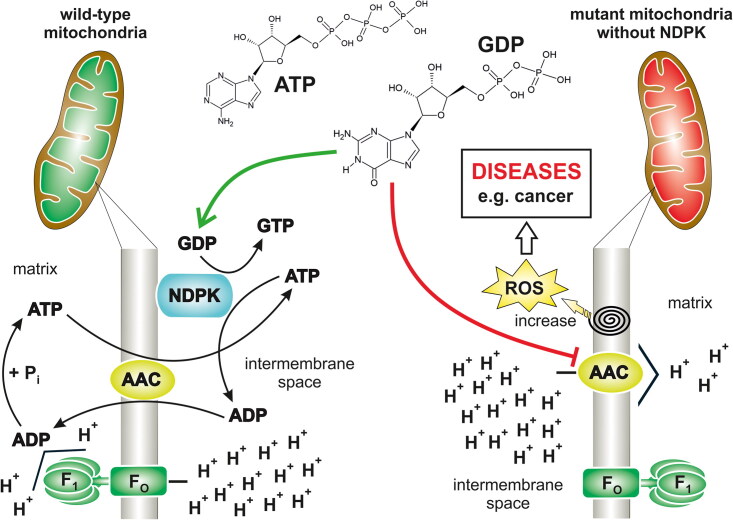

## Introduction

In a process known as proton (H^+^) leak or H^+^ conductance, the H^+^ translocation across the inner mitochondrial membrane (IMM) from the intermembrane space to the mitochondrial matrix, which bypasses F_O_F_1_-ATP synthase [F_O_ and F_1_ are two structurally and functionally distinct segments of ATP (adenosine 5′-triphosphate) synthase], constitutes an energy dissipation mechanism of uncoupling^1^^,^^2^. In contrast, the energy-conserving pathway in eukaryotes is primarily sustained by the mitochondrial F_O_F_1_-ATP synthase (also known as respiratory complex V), which usually and mainly exhausts the electrochemical H^+^ gradient generated by pumps of the respiratory chain (most often complexes I, III, and IV) to form vast amounts of ATP. Typically, the process of ATP molecule production coupled with oxygen-dependent respiration and driven by the oxidation of respiratory substrates is defined as oxidative phosphorylation (OXPHOS). In the IMM, in close proximity to F_O_F_1_-ATP synthase, an ADP(adenosine 5′-diphosphate)/ATP carrier (AAC) is embedded, which supervises ADP/ATP cycling in the cell^3^. Although it is generally known that AAC is primarily employed in the antiporting of some adenine nucleotides, the fact that this carrier is an important catalyst of “futile” H^+^ uptake in mitochondria, thus uncoupling, is not always mentioned^4–7^. It has been proposed that long-chain free fatty acids (FFAs), such as arachidonic acid and palmitic acid, may initiate AAC uncoupling activity, but there is no consensus regarding the actual mechanism, e.g. the FFA allosteric/cofactor mechanism^6^^,^^8^ or the FFA cycling mechanism^5^^,^^6^^,^^9^, which are often considered. These mechanisms are also proposed for uncoupling protein (UCP), i.e. another H^+^ carrier, often considered the primary catalyst of H^+^ leak in the IMM, which is commonly found in many organisms across eukaryotes^1^. The negative regulation of protein-mediated H^+^ leak needs to be finely adjusted to prevent ATP depletion in the cell. According to some results, guanosine 5′-diphosphate (GDP), usually at higher concentrations, i.e. 0.5–1 mM, could play the role of a direct and natural inhibitor of AAC-sustained H^+^ transport (based on different approaches)^9–13^, which also coincides with negative regulation of UCP^1^. The GDP-binding site of AAC/UCP faces the intermembrane space of the mitochondria.

Short-circuiting of H^+^ chemiosmosis in mitochondria through H^+^ leak is an innate energy dissipation mechanism that uncouples OXPHOS. Simply put, the phenomenon of OXPHOS is deprived of its maximal capacity and never reaches a potential 100% yield. However, for example, AAC-mediated unidirectional H^+^ transfer beyond F_O_F_1_-ATP synthase, which undeniably decreases the efficiency of ATP synthesis during aerobiosis, may be surprisingly beneficial. Mild (slight) uncoupling, including AAC-perpetrated H^+^ leak, allows for the maintenance of the redox balance of the electron transport chain, thus eliminating the risk of excess release of radical species, such as superoxide, which is classified as a reactive oxygen species (ROS)^1^^,^^7^. Therefore, partial uncoupling may be vital in counteracting oxidative stress, as evidenced, e.g. by elevated ROS levels. Moreover, controlled uncoupling may decrease the risk of the development of certain human diseases, such as cancer and obesity^9^^,^^14^^,^^15^. Effective cancer treatment may rely on the selective induction of apoptotic cell death, e.g. through a decrease in ATP levels. In contrast, effective obesity treatment could be achieved by modulating the metabolic rate and increasing energy expenditure, followed by weight loss, as an imitation of physical exercise.

**Figure 1.**
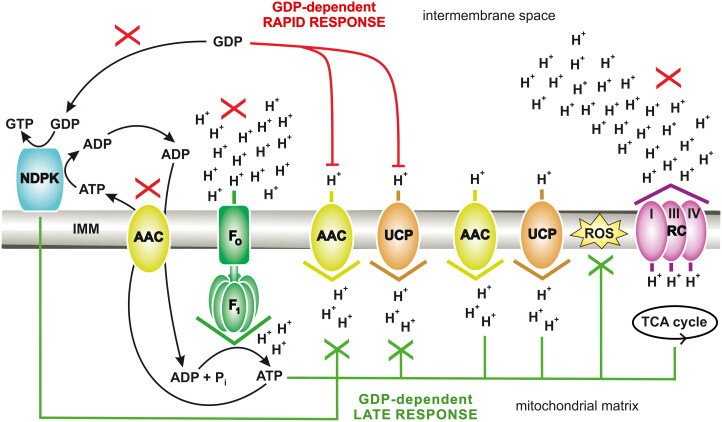
General model including the GDP function for energy transduction in mitochondria. The red “X” indicates nucleotide metabolism dysfunction, including any mtNDPK and oxidative phosphorylation (OXPHOS) disturbances, such as mild intoxication by carboxyatractyloside, and thus not total inhibition of the AAC, mtNDPK, or OXPHOS apparatus. Under such conditions, GDP provides a rapid response, which immediately counteracts AAC/UCP-mediated H^+^ leak (GDP causes a decrease in mild uncoupling *via* direct inhibition of AAC and UCP from the intermembrane space); this response is symbolised by red lines. In contrast, the green lines symbolise the GDP-dependent late response (direct and indirect), i.e. stabilisation of energy status, providing the balance between energy conservation required to survive and energy dissipation to avoid excessive release of free radicals in cells. A green “X” indicates downregulation, including both direct and indirect GDP-dependent late responses. The direct response involves the continuation of GDP-dependent partial inhibition of AAC/UCP-mediated H^+^ leak from the intermembrane space, whereas the indirect response allows for limited ROS release through partial uncoupling facilitated by AAC/UCP-mediated H^+^ leak. AAC: ADP/ATP carrier; F_O_F_1_: F_O_F_1_ ATP synthase; IMM: inner mitochondrial membrane; NDPK: nucleoside diphosphate kinase; P_i_: inorganic phosphate; RC: respiratory chain; ROS: reactive oxygen species; TCA cycle: tricarboxylic acid cycle; UCP: uncoupling protein; I, III, and IV: three H^+^-pumping complexes of RC. The figure was created using CorelDRAW.

AAC not only colocalizes with F_O_F_1_-ATP synthase^3^ but is also adjacent to a mitochondrial nucleoside diphosphate kinase (mtNDPK), which is numerically classified as EC 2.7.4.6 (Figure 1)^16^^,^^17^. Therefore, mtNDPK must directly influence OXPHOS and AAC activities (ADP/ATP turnover and H^+^ uptake). In mammals, this type of enzyme is often referred to as Nm23 (nonmetastatic clone 23) or NME (nucleotide metabolism enzyme), e.g. Nm23-H4 (NME4 or NDPK-D), which is the only human isoform within mitochondria (as a peripheral membrane protein attached to both sides of the IMM) directed to these organelles *via* a true mitochondrial targeting sequence^16^^,^^18^. In the case of yeast, e.g. *Saccharomyces cerevisiae*, the acronym Ynk1p or Ynk1 was coined for this kinase^19–21^, which homologs/isoforms generally exist in a hexameric state^16^. NDPKs are transphosphorylases with broad substrate specificity and proceed with the reactions of phosphate group exchange between nucleoside triphosphates, which are donors, and nucleoside diphosphates, which are acceptors, e.g. ATP + GDP → ADP + GTP (guanosine 5′-triphosphate) (Figure 1); this exchange occurs by a “ping-pong” mechanism involving phosphoenzyme intermediate, where conserved histidine undergoes autophosphorylation and Mg^2+^ (magnesium) ions in complex with nucleotides are crucial^22–25^. This ubiquitous kinase with different subcellular locations is considered an essential element of, for instance, purine nucleotide/energy channelling, namely, the distribution of particular substrates to many specialised microcompartments within the cell, forming intracellular units^18^. Regeneration of nucleoside triphosphates such as GTP, CTP, or UTP from corresponding nucleoside diphosphates often requires NDPK, which consumes ATP as its primary substrate. Generally, NDPKs use a wide range of secondary nucleotide substrates, often with the highest preference for guanosine diphosphate substrates. In some organisms, hexamers of NDPK are important local suppliers of GTP molecules to dynamins (including dynamin-related or dynamin-like proteins), which, among other functions, promote fission and fusion processes in mitochondria. Importantly, different NDPKs and dynamin superfamily proteins are considered proximate functional partners engaged in membrane remodelling and trafficking. As nucleoside triphosphates are also precursors for nucleic acid biosynthesis, it should be unsurprising that NDPK (the last enzyme in some nucleoside triphosphate synthesis pathways) influences DNA/RNA formation, thus influencing the cell cycle, including in yeast^23^^,^^26^^,^^27^. Disturbances in nucleoside triphosphate turnover, such as those resulting from temperature-dependent NDPK activity in yeast (*Schizosaccharomyces pombe*), may affect the rate of DNA replication^27^. Interestingly, downregulation and loss-of-function mutations in mammalian NDPK-D may cause an increase in ROS levels accompanied by an increase in lipid peroxides^28^, which coincides with a disbalance in mitochondrial uncoupling^1^. Understanding the biological context of NDPK homologs and isoforms, including those cooperating with mitochondria, is very important because these enzymes may exert pro- and antioncogenic effects, where the expression and activity of mitochondrial NDPK could be negatively associated with the progression of some cancer types and thus metastasis^28–32^. The molecular proximity of AAC to NDPK is certainly not indifferent. The combined downregulation of some AAC isoforms (which transport ATP produced by glycolysis into mitochondria) and inhibition of certain mitochondrial respiratory chain complexes, thereby targeting OXPHOS disturbances, may counteract tumorigenesis^33^.

**Figure 2.**
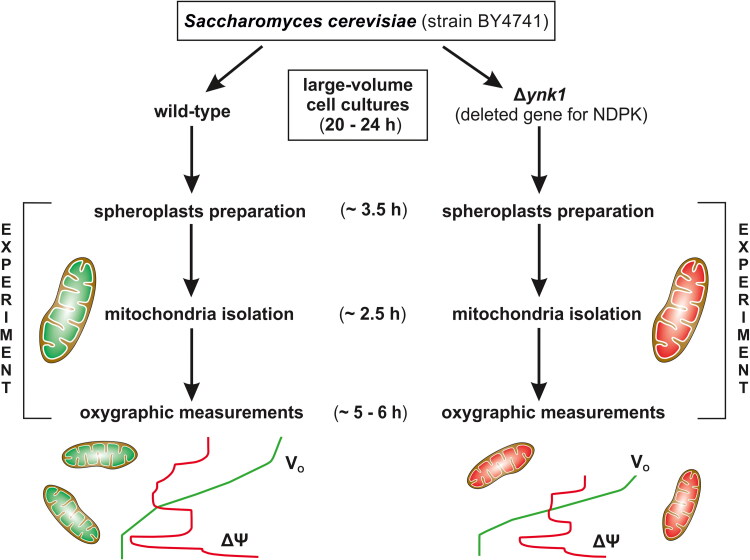
Schematic representation of the Materials and methods described in detail in the main text. Generally, the cell culture and mitochondria isolation protocols were identical for both types of yeast strains, i.e. wild-type strain and mutant strain without NDPK (nucleoside diphosphate kinase). Moreover, cell cultures, followed by spheroplast preparations and mitochondria isolations and then oxygraphic measurements, were carried out simultaneously or sequentially for each strain or preparation derived from it. Starting from spheroplast preparation, all subsequent steps were performed on the same day. V_O_: mitochondrial respiratory rate, ΔΨ: inner mitochondrial membrane electrical potential. The figure was created using CorelDRAW.

This work presents a novel concept, i.e. the crosstalk between mtNDPK, AAC, and OXPHOS and H^+^ leak in mitochondria. Specifically, the mutual impact of mtNDPK and different elements, which are responsible for the transport of electrons (respiratory chain), the transport of H^+^ (respiratory chain and H^+^ carriers, such as AAC), and the transport of nucleotides (AAC) to sustain aerobic respiration for survival (Figure 1). This cooperation was revealed by harnessing selected cell cultures and bioenergetic techniques, i.e. the use of tools such as specific electrodes to monitor, simultaneously, changes in the mitochondrial respiratory rate (V_O_) and thus in oxygen consumption (reduction in mitochondria *via* the respiratory chain) over time as well as changes in the inner mitochondrial membrane electrical potential (ΔΨ) (Figure 2). *S. cerevisiae* yeast cells have been used in studies, as the classic NDPK of this unicellular organism is probably encoded by a single gene, but its product is located both in the cytosol and mitochondria^19^^,^^20^^,^^26^. Interestingly, this yeast species is also naturally deficient in UCP^34^. Consequently, the yeast-based system eliminates the negative background resulting from the distribution of NDPK isoforms, e.g. in mammals^16^^,^^18^, and other major mitochondrial H^+^ leak catalysts, such as mammalian isoforms of UCP^1^. Therefore, the yeast model is appropriate for demonstrating the paramount importance of mtNDPK in regulating AAC-associated H^+^ leak alone. Notably, yeast mutants without NDPK, i.e. cells of the Δ*ynk1* deletion strain BY4741, were tested to determine the alternative destination of the mitochondrial GDP pool for respiratory chain activity, namely, the earlier proposed GDP-dependent inhibition of uncoupling *via* AAC^1^. NDPK was not detected in mitochondria isolated from such mutants^20^, which hitherto had never been used during bioenergetic studies aimed at OXPHOS naturally affected by H^+^ leak. Currently, commercially available yeast cells derived from wild-type and Δ*ynk1* strains are likely the best eukaryotic system for testing the crosstalk between mtNDPK, AAC, and OXPHOS and H^+^ leak in mitochondria. Notably, different NDPK functions have been found to be evolutionarily conserved across eukaryotes^18^. The details of rapid GDP metabolism and some factors negatively influencing its physiological pathways in mitochondria isolated from wild-type yeast, including the lack of apparent GDP metabolism in mutant mitochondria completely devoid of classic NDPK, have never been described before. Accordingly, in the bioenergetic context, this is the first attempt to provide information concerning a unique biological model for creating a system with and without NDPK. Moreover, while not a common practice in bioenergetics, the *in vitro* approach using media that facilitate OXPHOS, regardless of the nucleotide type used during oxygraphic measurements aimed at H^+^ leak, more accurately reflects physiological aspects of nucleotide metabolism in the mitochondria. Therefore, this work presents a new perspective on the regulation of the energy transduction process *via* mitochondrial OXPHOS, which is generally applicable to all eukaryotes. Finally, the two-sided activity of AAC, ADP/ATP transport versus H^+^ transport^5^^,^^6^^,^^8^ is explored in greater depth, which increases the general understanding of bioenergetics in the context of some diseases, including metabolic disorders such as obesity and cancer^9^^,^^14^^,^^15^^,^^33^.

## Materials and methods

### Yeast strains and culture conditions

Commercially available *S. cerevisiae* haploid strains, i.e. (i) wild-type BY4741 (*MATa*; *ura3Δ0*; *leu2Δ0*; *his3Δ1*; *met15Δ0*) and (ii) the BY4741 mutant (Δ*ynk1*) with a deleted gene for NDPK (*MATa*; *ura3Δ0*; *leu2Δ0*; *his3Δ1*; *met15Δ0*; *YKL067w*::kanMX4), were used [Euroscarf, Germany]. Cells derived from both strains were grown simultaneously at 28 °C under vigorous aeration (180 rpm) in four stages. Specifically, the sterile cultures started from solid YPD medium (1% yeast extract, 1% Bacto Peptone, 2% glucose, and 2% agar) and, after 20–24 h, were followed by composition-varied small-volume (25 ml) liquid media, i.e. first YPD medium (1% yeast extract, 2% Bacto Peptone, and 2% glucose) and then YPG medium (1% yeast extract, 2% Bacto Peptone, and 3% glycerol). Each such culture was also carried out within 20–24 h. Finally, the single preculture was transferred to a large-volume (850 ml) YPG liquid medium. In total, four large-volume cultures for each strain were likewise grown out for up to 24 h. The growth rate of the yeast cells derived from the BY4741 mutant strain was lower than that of the congenic yeast cells of the BY4741 wild-type strain, but the viability of the mutants allowed it to isolate the mitochondria effectively. Yeast cell growth was monitored by measuring the optical density (OD) at 546 nm (Shimadzu UV-1650PC, Japan). The generation times in continuously agitated cultures were approximately 3 h and 4.5 h for cells of the wild-type and Δ*ynk1* strains, respectively.

### Preparation of yeast spheroplasts

According to a previously established procedure with modifications^35^, spheroplasts were prepared simultaneously from cells derived from the wild-type and Δ*ynk1* strains. Specifically, *S. cerevisiae* cells were harvested by centrifugation (3,000×*g*, 10 min, 4 °C) in the exponential (logarithmic) growth phase at an OD (λ = 546 nm) of approximately 2.9 for cells of the wild-type strain and 1.7 for cells of the Δ*ynk1* mutant strain, maximally after 24 h of large-volume (850 ml) YPG liquid medium culture. The spheroplast preparation was started early in the morning to complete the subsequent steps (mitochondria isolation and oxygraphic measurements) within one day. The cell pellets, usually approximately 8 g for the wild-type strain and 5 g for the Δ*ynk1* mutant strain, were rinsed twice with bidistilled water (3,000×*g*, 5 min, 4 °C). The cells were subsequently resuspended in buffer containing 0.1 M Tris-HCl and 10 mM dithiothreitol (1 g, wet weight, per 6 ml) and incubated at 28 °C (15 min) with orbital shaking (125 rpm). Then, these suspensions were again centrifuged (3,000×*g*, 5 min, 4 °C), washed once with a cold solution of 1.2 M sorbitol (3,000×*g*, 10 min, 4 °C), and resuspended in an enzymatic digestion buffer (room temperature) based on 1.2 M sorbitol and containing 20 mM KH_2_PO_4_/K_2_HPO_4_ and 10 mM Tris-HCl, again at 1 g of cells (wet weight) per 6 ml. Subsequently, 100 U of Zymolyase-100T was added per 1 g of cells (wet weight). The suspensions with lytic enzymes were incubated at 28 °C under gentle agitation (125 rpm). The degradation of the yeast cell walls was monitored spectrophotometrically (at λ = 546 nm) as the OD decreased. When 83–85% of the cells had been converted into spheroplasts (usually 30–35 min), the suspensions were centrifuged (3,000×*g*, 10 min, 4 °C). Finally, after lyticase treatment, spheroplasts were harvested and subsequently rinsed twice with ice-cold 1.2 M sorbitol (3,000×*g*, 10 min, 4 °C). Except for incubation steps at 28 °C and depending on needs, the procedure was performed on ice.

### Isolation of yeast mitochondria

Mitochondria were isolated by differential centrifugation in a cold room (6–8 °C) on ice and in refrigerated buffers, according to previously established procedures with modifications^35^, simultaneously from freshly prepared spheroplasts derived from cells of the wild-type and Δ*ynk1* strains, usually from approximately 7 g and 4 g, respectively. Spheroplasts were resuspended in buffer (pH = 7.4) containing 0.65 M mannitol with 20 mM Tris-HCl supplemented with 0.1% bovine serum albumin (BSA), 0.5 mM EDTA, 0.1 mM EGTA, and 1 mM PMSF (protease inhibitor) at proportion of 1 g (wet weight) per 6 ml. This suspension was homogenised on ice in a Dounce device, usually by 12–14 strokes (down and up) with a tight-fitting plunger. The presence of BSA in the isolation medium allowed the endogenous free fatty acids (e.g. activators of AAC-mediated mitochondrial H^+^ leak) to be chelated from the suspension. The homogenate was filtered through sterile gauze, and the filtrate was centrifuged at 1,000×*g* for 10 min at 4 °C. The supernatant was then centrifuged at 10,000×*g* for 10 min at 4 °C to sediment the crude mitochondria. Another low-speed centrifugation (1,000×*g*, 10 min, 4 °C) of resuspended pellets (to remove residual cell debris) preceded the next high-speed centrifugation of supernatants at 10,000×*g* (10 min, 4 °C), both in buffer (pH = 7.4) based on 0.65 M mannitol with 20 mM Tris-HCl supplemented only with 0.1% BSA and 0.2 mM EGTA. To achieve the highest purity of mitochondria, the organellar pellet was additionally rinsed twice by resuspension and recentrifuged under the same high-speed conditions. Finally, the isolated mitochondria were resuspended in ice-cold buffer (pH = 6.9), which generally contained 0.2% BSA, 0.5 mM EGTA, 10 mM HEPES, 5 mM KCl, 10 mM KH_2_PO_4_/K_2_HPO_4_, 0.65 M mannitol, and 2 mM MgCl_2,_ to obtain a concentration of approximately 25 mg of protein/ml for cells of the wild-type strain and 15 mg of protein/ml for cells of the Δ*ynk1* mutant strain. The biuret method was used to determine the mitochondrial protein concentration with BSA as a standard.

### Measurement of mitochondrial respiration, oxygen uptake, and membrane potential

**Figure 3.**
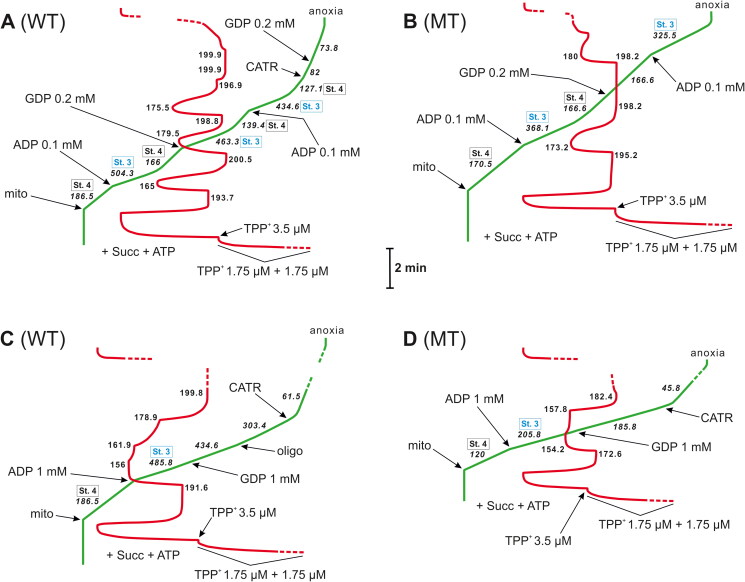
The 0.1 or 1 mM ADP-induced OXPHOS was followed by different effects of GDP (0.2 or 1 mM) depending on the type of isolated mitochondria. An illustrative record of V_O_ (green) and ΔΨ (red) measurements, initially in the absence of OXPHOS inhibitors. The ATP concentration was 0.35 mM in the presence of 0.1 mM ADP and 0.2 mM GDP (**A** and **B**) or 2 mM in the presence of 1 mM ADP/GDP (**C** and **D**). ATP was added before energisation with succinate (Succ, 5 mM), which served as the oxidisable substrate. ADP and GDP were added after energisation. When added, carboxyatractyloside (CATR) was used at 3.6 µM and oligomycin (oligo) was used at 2 µg. Calibration of the electrode for ΔΨ measurement with approximately 7 µM TPP^+^ in total (with three sequential additions, i.e. 1.75 μM, 1.75 μM, and 3.5 μM). WT: mitochondria isolated from wild-type yeast. MT: mitochondria isolated from Δ*ynk1* yeast without NDPK. The numbers on the traces refer to O_2_ consumption rates (V_O_ in italics) in nmol O/min/mg of mitochondrial protein (mito) or to the ΔΨ values in mV. St. 4 (black boxes adjacent to the V_O_ values but also attributed to the corresponding ΔΨ values) refers to State 4, thus, nonphosphorylating respiration measured in the absence of exogenous ADP or GDP, and St. 3 (blue boxes adjacent to the V_O_ values but also attributed to the corresponding ΔΨ values) refers to State 3, thus, phosphorylating respiration directly induced by ADP or indirectly by GDP. State 4 and State 3 were recorded in the absence of any exogenous OXPHOS inhibitors. The traces shown are from representative measurements, which were carried out under conditions described in detail in the Materials and methods. The figure was created using CorelDRAW.

Molecular physiology experiments based on bioenergetic approaches were carried out with freshly prepared mitochondria, which constituted the last step of laboratory work on the same day. Oxygen uptake was measured polarographically using a Clark-type oxygen electrode (Rank Brothers, UK) in 2.8 ml of incubation medium (such as that used for the final mitochondrial resuspension, Section “Isolation of yeast mitochondria”) at 28 °C (in a water-jacketed chamber), usually with approximately 0.8–1 mg of mitochondrial protein. The V_O_ values are given in nanomoles of oxygen atoms per minute per milligram (nmol O/min/mg) of mitochondrial protein. The ΔΨ was measured simultaneously with O_2_ consumption using a tetraphenylphosphonium (TPP^+^)-specific electrode, as previously described, but with some modifications^36^. This electrode was calibrated with three sequential additions of TPP^+^, i.e. approximately 1.75 μM, 1.75 μM, and 3.5 μM. The relative values of ΔΨ changes are given in millivolts (mV). Succinate was used as an oxidisable substrate (5 mM). Commonly used bioenergetic coupling parameters, i.e. the ADP/O ratio and respiratory control ratio (RCR), were calculated as previously described^37^. The ADP pulse method was used to determine the ADP/O ratio of OXPHOS. The quotient of nmol of ADP (added exogenously), in this study, approximately 280 nmol of ADP (0.1 mM) (Table 1; Figure 3A,B), and the total amount of oxygen atoms (nmol) consumed in mitochondria during State 3 respiration constituted the ADP/O ratio. Therefore, the ADP/O ratio reflects the number of ATP molecules synthesised in mitochondria from ADP and inorganic phosphate for each oxygen atom consumed during OXPHOS^2^^,^^38^. In turn, RCR is equal to the quotient of State 3 V_O_ and State 4 V_O_ following State 3 (State 3 V_O_/State 4 V_O_) and reveals the tightness of OXPHOS^37^. State 3, preceded and followed by State 4 (Table 1; Figure 3A,B), refers to phosphorylating respiration, usually measured in the presence of exogenous ADP but in the absence of any exogenous OXPHOS inhibitors. In this work, State 3 was also indirectly induced by GDP (0.2 or 1 mM) *via* mtNDPK. State 4 refers to nonphosphorylating respiration, measured in the absence of exogenous ADP or GDP and in the absence of exogenous OXPHOS inhibitors. State 4 respiration is generally accepted to drive H^+^ leak^4^. State 3 of aerobic respiration, initiated by the addition of ADP (or its generation *via* mtNDPK from ATP), lasts as long as ADP is available for OXPHOS, and when the entire ADP pool is exhausted and thus transformed to ATP *via* F_O_F_1_-ATP synthase, State 3 transitions again to State 4 (analogous to State 4 before ADP addition), as presented in Figure 3A,B. High-quality mitochondrial preparations, i.e. with values of ADP/O approximately 1.1 and RCR around 3.2–3.3 (Table 1), were best fitted for experiments. The coupling parameters of yeast can be lower than those of mammals, as ATP, generated after ADP-induced OXPHOS, may contribute to H^+^ permeability through the IMM^39^^,^^40^.

**Table 1.** Selected effects of ADP and GDP on respiration and coupling parameters in mitochondria isolated from yeast cells of wild-type and Δ*ynk1* strains.

**Wild-type yeast**						**Yeast mutant without NDPK** (Δ*ynk1*)			
**Basal State 4** (+ 2 mM ATP)						**Basal State 4** (+ 2 mM ATP)		**State 4** (+ 2 mM ATP) **+ 1 mM GDP**	
V_O_ = 195 ± 7.6 ΔΨ = 193.9 ± 1.1						V_O_ = 156.8 ± 8.8 ΔΨ = 182.7 ± 2.8		V_O_ = 139.2 ± 14.2 ΔΨ = 183 ± 3.5	
**State 3**		**State 3**		ADP/O*	RCR*	**State 3**		ADP/O*	RCR*
**GDP 0.2 mM** (+ 0.35 mM ATP)	**GDP 1 mM** (+ 2 mM ATP)	ADP* 0.1 mM (+ 0.35 mM ATP)	ADP 1 mM (+ 2 mM ATP)			ADP* 0.1 mM (+ 0.35 mM ATP)	**ADP 1 mM** (+ 2 mM ATP)		
V_O_ = 279.4 ± 49.6 ΔΨ = 190.5 ± 3.1	**MEGA**V_O_ = 443.2 ± 65.4 ΔΨ = 170.6 ± 3.9	V_O_ = 479.6 ± 43.9 ΔΨ = 165.9 ± 3.9	V_O_ = 500.3 ± 28.1 ΔΨ = 161.8 ± 2.9	1.1 ± 0.05	3.2 ± 0.1	V_O_ = 327.3 ± 45.4 ΔΨ = 161.8 ± 5.5	V_O_ = 228.3 ± 40.3 ΔΨ = 155.1 ± 5.3	1.1 ± 0.05	3.3 ± 0.7
	**STANDARD**V_O_ = 243.5 ± 11.7 ΔΨ = 189.8 ± 1.3								

Basal State 4 refers to the initial (after energisation with 5 mM succinate, which served as the oxidisable substrate) nonphosphorylating respiration measured in the absence of exogenous ADP or GDP but in the presence of ATP (0.35 or 2 mM). In the case of wild-type and mutant mitochondria, basal State 4 respiration in the presence of 0.35 mM ATP (not shown) was similar to the basal State 4 respiration in the presence of 2 mM ATP. ATP was added before energisation, but ADP and GDP were added after energisation. State 3 refers to phosphorylating respiration, measured in the presence of exogenous ADP (0.1 or 1 mM) or GDP (0.2 or 1 mM). GDP-mediated State 3 is characteristic of only wild-type mitochondria with mtNDPK, where 0.2 or 1 mM GDP indirectly (*via* mtNDPK) stimulates OXPHOS (increase in V_O_ and decrease in ΔΨ); thus, a transition of State 4 in State 3 is observed. In Δ*ynk1* mitochondria without mtNDPK, GDP is not stimulatory; therefore, 1 mM GDP has a recoupling effect (decrease in V_O_ and increase in ΔΨ), and there is no transition of State 4 in State 3. In turn, ADP (0.1 or 1 mM) directly stimulates OXPHOS in both types of mitochondria (wild-type and Δ*ynk1*). In the present study, State 4 and State 3 were recorded in the absence of any exogenous OXPHOS inhibitors. * refers to conditions with 0.1 mM ADP, which allowed the determination of the coupling parameters ADP/O and RCR. The results are presented as the mean values ± standard error (SE), which were obtained under conditions described in detail in the Materials and methods.

### OXPHOS, NDPK activity, and H^+^ leak measurements

Generally, traces were generated in the same essential medium as that used to prepare a final mitochondrial suspension (Section “Isolation of yeast mitochondria”), which was verified as optimal for oxygraphy. The starting medium was supplemented with 0.35 mM ATP to determine the coupling parameters (ADP/O and RCR) with a pulse of ADP (0.1 mM) or GDP (0.2 mM). However, 2 mM ATP was used to test the influence of 1 mM GDP on NDPK activity and H^+^ leak. In the presence of 2 mM ATP, other nucleotides, i.e. ADP, GDP, and GTP, were administered at a 1 mM concentration to maintain a 1:2 ratio with ATP. A concentration of ATP reaching 2 mM can be considered a subphysiological threshold for the yeast *S. cerevisiae*^41^^,^^42^. The presence of exogenous ATP (0.35 or 2 mM) not only promoted the stimulatory effect of GDP (0.2 or 1 mM) but was also needed to obtain a steady State 4 respiration energised with succinate. In the absence of supplementary ATP, V_O_ and ΔΨ were unstable, and both parameters decreased with time. Free ATP (not complexed with Mg^2+^) may promote substrate oxidation in isolated mitochondria because this purine nucleotide was identified, among other functions, as a positive effector for terminal cytochrome *c* oxidase in the respiratory chain, including yeast enzymes^40^^,^^42–44^.

To date, most functional studies of mitochondrial H^+^ leak (AAC- and UCP-mediated) have been performed in the presence of oligomycin (an inhibitor of F_O_F_1_-ATP synthase that prevents OXPHOS) and thus under State 4, which cannot transition into State 3^5–8^^,^^10–12^^,^^43^^,^^45–49^. However, these types of assays do not reflect physiological conditions under which State 3 and State 4 of mitochondrial respiration coexist. Generally, the traces of initial mitochondrial respiration presented here were recorded without the typically used OXPHOS inhibitors, i.e. oligomycin and carboxyatractyloside (CATR, an inhibitor of AAC). These xenobiotics were only administered acutely (after energisation).

### Statistical analysis

The results are presented as the mean values ± standard error (SE) obtained from at least five separate mitochondrial isolations (from different experiments on different days), with determinations performed at least in duplicate whenever possible. Results expressed as percentages are relative to the corresponding controls established for preparations derived from cells of the wild-type and Δ*ynk1* strains. Comparisons were made using Student’s *t*-test (an unpaired two-tailed test) to evaluate the significance of the difference between the control and treated measurements. Differences were considered statistically significant if *p* < 0.1 (*), *p* < 0.01 (**), or *p* < 0.001 (***).

### Chemicals

Reagents for bioenergetic measurements, including nucleotides (ADP, ATP, GDP, and GTP), inhibitors (e.g. CATR), oxidisable substrate (succinate), different salts (e.g. MgCl_2_), dithiothreitol, EDTA, EGTA, mannitol, sorbitol, and buffering agents (e.g. HEPES), as well as BSA, were obtained from Sigma–Aldrich (MO, USA). These solutions were prepared with bidistilled water. Oligomycin and PMSF were initially dissolved in methanol. Stock solutions of nucleotides were adjusted to a pH of approximately 7.0 with KOH. Agar, Bacto Peptone, and yeast extract were obtained from BD Bioscience (CA, USA). Zymolyase-100T was obtained from Nacalai Tesque (Kyoto, Japan).

## Results

### GDP metabolism via mtNDPK coincides with the ADP stimulatory effect

Regardless of the measurement conditions, i.e. in the absence or presence of exogenous FFAs, AAC-mediated H^+^ leak is probably always positively dependent on protonatable FFAs^8^^,^^9^. According to this view, so-called “basal” H^+^ conductance, which is usually registered in the presence of BSA controlling the FFA content in the medium without the addition of exogenous FFAs, is, in fact, influenced by endogenous FFAs. Therefore, “constitutive” protein-sustained H^+^ leak, which is generally AAC- and/or UCP-mediated, should not be seen as unregulated and dependent only on the presence of specific carrier proteins, as proposed previously^4^. Total depletion of FFAs from mitochondrial preparations, especially those residing in membranes, which are accessible to AAC and other H^+^ leak catalysts, is challenging. Mitochondrial phospholipases may permanently release FFAs for various purposes (both *in vivo* and *in vitro*), including the stimulation of nonphosphorylating H^+^ translocation^8^. Notably, the studies presented here were carried out in model organisms naturally lacking UCP and in the absence of exogenous FFAs but in the presence of BSA. Distinctly, earlier findings indicate that FFAs, after being transformed to long-chain fatty acyl-coenzyme A, such as palmitoyl-CoA (P-CoA) (Section “OXPHOS inhibitors suppress mtNDPK activity and remodel GDP function”), or anionic phospholipids, such as cardiolipin, may contribute to the inhibition of NDPK phosphotransfer activity^29^^,^^50^.

**Figure 4.**
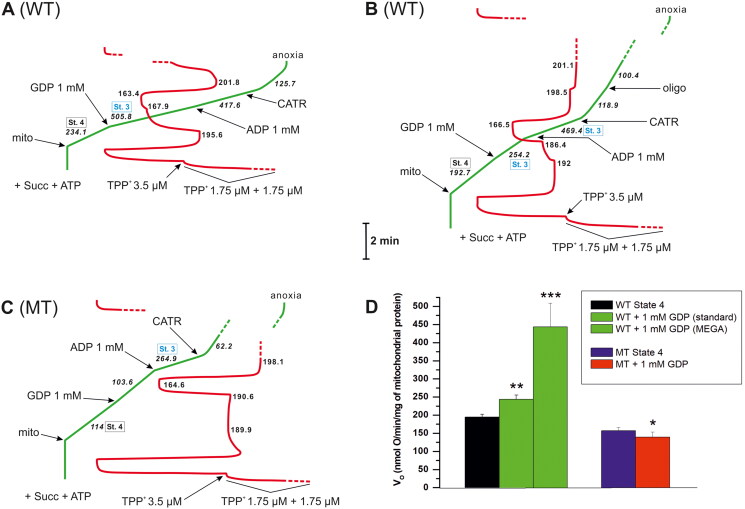
Different effects of 1 mM GDP were followed by different effects of 1 mM ADP depending on the type of isolated mitochondria. An illustrative record of V_O_ (green) and ΔΨ (red) measurements, initially in the absence of OXPHOS inhibitors. Succinate (Succ, 5 mM) served as the oxidisable substrate in the presence of 2 mM ATP, which was added before energisation. ADP and GDP were added after energisation. Mega 1 mM GDP effect (**A**), standard 1 mM GDP effect (**B**), and lack of GDP stimulatory effect in mitochondria isolated from mutant cells without NDPK (**C**). The changes in V_O_ (**D**) registered after 1 mM GDP addition for the tested conditions with mean values ± standard errors (SE), WT State 4: V_O_ = 195 ± 7.6; WT + 1 mM GDP (standard): V_O_ = 243.5 ± 11.7; WT + 1 mM GDP (MEGA): V_O_ = 443.2 ± 65.4; MT State 4: V_O_ = 156.8 ± 8.8; MT + 1 mM GDP: V_O_ = 139.2 ± 14.2. Standard and mega 1 mM GDP effects are indirectly (*via* mtNDPK) coupled with OXPHOS; therefore, 1 mM GDP is an indirect inducer of State 3. When added, carboxyatractyloside (CATR) was used at 3.6 µM and oligomycin (oligo) was used at 2 µg. Calibration of the electrode for ΔΨ measurement with approximately 7 µM TPP^+^ in total (with three sequential additions, i.e. 1.75 μM, 1.75 μM, and 3.5 μM). WT: mitochondria isolated from wild-type yeast; MT: mitochondria isolated from Δ*ynk1* yeast without NDPK. The numbers on the traces refer to O_2_ consumption rates (V_O_ in italics) in nmol O/min/mg of mitochondrial protein (mito) or to the ΔΨ values in mV. St. 4 (black boxes adjacent to the V_O_ values but also attributed to the corresponding ΔΨ values) refers to State 4, thus, nonphosphorylating respiration measured in the absence of exogenous ADP or GDP, and St. 3 (blue boxes adjacent to the V_O_ values but also attributed to the corresponding ΔΨ values) refers to State 3, thus, phosphorylating respiration directly induced by ADP or indirectly by GDP. State 4 and State 3 were recorded in the absence of any exogenous OXPHOS inhibitors. The traces shown are from representative measurements, which were carried out under conditions described in detail in the Materials and methods. Differences were considered statistically significant if *p* < 0.1 (*), *p* < 0.01 (**), or *p* < 0.001 (***). The figure was created using CorelDRAW.

As mentioned earlier in this paper, it has been shown in some cases that GDP (usually at relatively high concentrations, i.e. 0.5–1 mM) could be a direct and natural inhibitor of AAC-mediated H^+^ transport^9–13^. In isolated mitochondria, such inhibition is revealed by a decrease in V_O_ accompanied by an increase in ΔΨ, usually (classically), in the presence of oligomycin, which inhibits OXPHOS^1^^,^^51^. However, in the absence of OXPHOS inhibitors (CATR, oligomycin, and P-CoA) but with supplementation with exogenous ATP (0.35 or 2 mM), the exogenously added GDP (0.2 or 1 mM) stimulates respiration of the isolated mitochondria of yeast (Figures 3A and 4A,B,D and Tables 1 and 2). Under these conditions, which can be termed physiological-like, the use of a nonclassic medium for H^+^ leak measurements has led to the proposal that mtNDPK effectively transforms GDP into GTP in mammals at the expense of ATP (ATP + GDP → ADP + GTP)^51–54^. Finally, a resulting ADP pool is, in fact, the reason for the observed increase in V_O_ and decrease in ΔΨ, indicating OXHPOS induction instead of H^+^ leak inhibition by GDP. Therefore, GDP should be seen as an indirect inducer of OXPHOS, which is necessary to regenerate free mtNDPK (GDP accepts phosphate previously donated to the kinase *via* ATP). Indeed, ATP causes a rapid transition of yeast NDPK into a phosphoenzyme^25^. However, considering the GDP stimulatory effect observed in this work, ADP (derived from exogenous ATP) had to be released from yeast mtNDPK *in vitro* under GDP pressure. Without exogenous GDP, OXPHOS is not exposed, as demonstrated by the stability of State 4 respiration in the presence of ATP (0.35 or 2 mM) alone (Figures 3 and 4, and Table 1).

**Table 2.** A descriptive and simplified summary of different effects of 1 mM GDP (revealed as changes in V_O_ and ΔΨ in isolated mitochondria, as described in the Results section), obtained in the presence of ATP (2 mM) and Mg^2+^ (2 mM) after energisation with succinate (5 mM), as outlined in the Materials and methods section.

**CONDITIONS** **+ ATP** **+ Mg^2+^**	**+ GDP 1 mM**
**wild-type yeast** no exogenous effectors limiting mtNDPK activity and OXPHOS	stimulatory effect **→** OXPHOS induction (indirectly *via* mtNDPK)
**wild-type yeast** + CATR	no stimulatory effect **→** H^+^ leak inhibition
**wild-type yeast** + P-CoA	no stimulatory effect **→** H^+^ leak inhibition
**yeast mutant without NDPK (Δ*ynk1*)**	no stimulatory effect **→** H^+^ leak inhibition

Carboxyatractyloside (CATR) was used at 3.6 µM, and palmitoyl-CoA (P-CoA) was used at 100 µM.

In the original studies presented here, three types of GDP stimulatory effects were distinguished in the mitochondria isolated from wild-type yeast. First, the low GDP concentration (0.2 mM) can cause the standard State 4 (before ADP/GDP addition) – State 3 (after ADP/GDP addition) – State 4 (when the ADP pool is exhausted) transitions typical for the ADP stimulatory effect (Figure 3A). Compared with 0.1 mM ADP-induced OXPHOS, which was manifested by RCR = 3.2 ± 0.1 and ADP/O = 1.1 ± 0.05 (Table 1), 0.2 mM GDP-induced OXPHOS revealed quite similar RCRs, i.e. 3.4 ± 0,1, and much higher GDP/O values, i.e. 2.3 ± 0.1. The apparently inflated GDP/O value could indicate that a GDP concentration greater than 0.2 mM is needed to release the entire, initially bound, pool of ADP (derived from dephosphorylated exogenous ATP) from yeast mtNDPK. Otherwise, partial dissociation of such ADP from mtNDPK gives overrated GDP/O, which, in fact, does not mirror the true ADP/O, i.e. the entire ADP pool generated *in situ* is not exhausted for ATP synthesis; the apparently indirectly induced State 3 respiration after GDP (0.2 mM) addition has shorter duration than State 3 after the addition of exogenous ADP (0.1 mM) alone. Unfortunately, the use of more GDP than 0.2 mM disabled the observation of State 4 – State 3 – State 4 transitions with GDP and calculation of RCR and GDP/O. However, even if the GDP/O is countable, it can be considered falsely magnified ADP/O.

In turn, the addition of 1 mM GDP to stabilised State 4 respiration with a saturated ATP concentration (2 mM) and a V_O_ = 195 ± 7.6 and ΔΨ = 193.9 ± 1.1 revealed two types of stimulatory effects in freshly isolated mitochondria from cells of the wild-type yeast strain (Table 1). Specifically, solely while recording the first trace, a robust response to 1 mM GDP was observed (Figure 4A; Table 1), resulting in V_O_ = 443.2 ± 65.4 and ΔΨ = 170.6 ± 3.9, thus a 227.3% increase in V_O_ and a 12% decrease in ΔΨ compared with the control conditions. The following measurements under the same conditions (technical repetitions), here collectively defined as the “standard 1 mM GDP effect”, always revealed a much weaker 1 mM GDP stimulatory effect in mitochondria isolated independently (Figure 4B; Table 1), i.e. V_O_ = 243.5 ± 11.7 and ΔΨ = 189.8 ± 1.3, resulting in a 124.9% increase in V_O_ and a 2.1% decrease in ΔΨ. Therefore, the original and more pronounced changes in V_O_ and ΔΨ induced by 1 mM GDP were termed here the “mega 1 mM GDP effect” elicited by this purine nucleotide, which resembled a pure 1 mM ADP-induced OXPHOS with V_O_ = 500.3 ± 28.1 and ΔΨ = 161.8 ± 2.9, i.e. a 256.6% increase in V_O_ and a 16.5% decrease in ΔΨ compared with the control conditions (Figure 3C; Table 1). The mega 1 mM GDP effect can be explained by the additional and endogenous ADP pool deposited in yeast mtNDPK, which was gradually released upon the first exogenous GDP injection (1 mM). Within a short time (the second trace was usually started 15–30 min after the first trace), this ADP pool in mitochondrial preparations could be spontaneously discharged and metabolised, thus excluding the repetition of the mega 1 mM GDP effect. The half-life of the yeast mtNDPK is a relatively negligible factor, as the standard 1 mM GDP effect can be quite stable within a few hours under the presented conditions (even over 5 h). In addition, NDPK is reported to have a half-life of approximately 6 h in cells^55^. Interestingly, 1 mM ADP added after the mega 1 mM GDP effect was not an effector of OXPHOS and ultimately contributed to respiratory chain inhibition, as V_O_ (329.2 ± 75.4) and ΔΨ (161.5 ± 7.9) simultaneously decreased, i.e. by 25.7% and 5.3%, respectively (Figure 4A). These findings support the idea that during the mega 1 mM GDP effect, mitochondria are at a full saturation level with ADP. Moreover, the negative outcome of the 1 mM ADP in this context may essentially result from a sudden and unbalanced increase in the local ATP level. Importantly, a sufficiently high ATP concentration (1–5 mM), which is in the physiological range, can effectively restrain yeast cytochrome *c* oxidase activity, and the action of ATP from the matrix side is considered^44^. Otherwise, 1 mM GDP added after 1 mM ADP counteracted OXPHOS and caused a decrease in V_O_ (445.2 ± 37.6), which was accompanied by an increase in ΔΨ (166.6 ± 2.4), i.e. by 11% and 103%, respectively (Figure 3C). However, 1 mM ADP administered after the standard 1 mM GDP effect could trigger OXPHOS (Figure 4B), and the ADP-dependent changes in V_O_ (387.9 ± 21.5) and ΔΨ (164.4 ± 1.9) in such a context were much more conspicuous than the preceding standard 1 mM GDP effect. However, when the effects of 1 mM ADP were compared, i.e. 1 mM ADP effect after the standard 1 mM GDP effect and the pure 1 mM ADP effect (no exogenous GDP), it was apparent that the presence of 1 mM GDP constituted a weakening factor of 1 mM ADP-induced OXPHOS. The presence of 1 mM GDP limited the 1 mM ADP-dependent increase in V_O_ accompanied by a decrease in ΔΨ, which resulted in a lower value of V_O_ and a higher value of ΔΨ for this type of 1 mM ADP addition (after 1 mM GDP). This finding clearly suggests that such a scenario is a consequence of the competitive/inhibitory effect of GDP exerted on AAC^10^^,^^12^, which can also be involved in GDP transport^56^.

In view of the evident stimulatory effect of 1 mM GDP (coupled with OXPHOS *via* mtNDPK) on mitochondria isolated from wild-type yeast (Figures 3A and 4A,B,D), as in mammalian preparations^51^, the supposedly innate GDP-dependent inhibition of H^+^ leak by blocking AAC uncoupling activity, as proposed earlier^9–13^, is once again called into question^51^, especially under conditions favouring OXPHOS. Intriguingly, other potential carrier/channel-type catalysts of futile H^+^ uptake embedded in the IMM of yeast are rather not inhibited by GDP. For example, in mitochondria isolated from yeast, in the presence of atractyloside, which is considered a weaker inhibitor of AAC antiport activity than CATR but binds at the same site^57^, and/or oligomycin, GDP (e.g. 1–2 mM) can also be an activating factor, i.e. it stimulates the permeability of the IMM, probably *via* the targeted opening of unspecific channels or the activation of undefined carriers^58^^,^^59^. This carrier/channel-dependent phenomenon could be the pathway for cation uniports (e.g. H^+^ translocation), thus contributing to an uncoupling effect on yeast mitochondrial respiration^39^^,^^40^^,^^59^. Therefore, overreduction of the respiratory chain, e.g. as the result of OXPHOS restriction, may sensitise mitochondria to given purine nucleotides (acting from the outer face of the IMM, thus from the intermembrane space) more probably towards opening of unspecific channels to maintain redox balance when minor energy demand occurs^40^^,^^58^^,^^60^. Such a short-circuiting pathway is often designated a yeast mitochondrial unselective channel or yeast permeability transition pore^61^. Importantly, not only GDP but also ATP and GTP can be robust activators of yeast mitochondrial permeability (seen as an intricate type of energy-dissipating pathway) *via* similar modes of action^39^^,^^40^^,^^58–62^. In contrast, the results of the experiments presented here, which were conducted under physiological-like conditions (without OXPHOS exclusion and with up to 2 mM exogenous ATP), revealed different effects of ATP and GDP. These nucleotides are interpreted mainly as substrates for mtNDPK (located in the mitochondrial intermembrane space) and both, indirectly *via* NDPK, stimulated OXPHOS. Therefore, ATP and GDP are involved in energy conservation rather than energy dissipation. Moreover, the magnitude of the stimulatory effects of 1 mM ADP (V_O_ = 500.3 ± 28.1 and ΔΨ = 161.8 ± 2.9) and standard 1 mM GDP (V_O_ = 243.5 ± 11.7 and ΔΨ = 189.8 ± 1.3) towards OXPHOS in the presence of 2 mM exogenous ATP were comparable with OXPHOS measured at 0.35 mM exogenous ATP and elicited by corresponding nucleotides at a lower concentration, i.e. 0.1 mM ADP (V_O_ = 479.6 ± 43.9 and ΔΨ = 165.9 ± 3.9) and 0.2 mM GDP (V_O_ = 279.4 ± 49.6 and ΔΨ = 190.5 ± 3.1) (Table 1). These findings may even exclude the concentration-dependent side effects resulting from the at least dual action of ATP and GDP, which operate from the outer face of the IMM. Additionally, it was previously shown that at 0.1 mM ADP (in the absence of OXPHOS inhibitors and exogenous ATP), the potentially negative effect of *in situ*-generated ATP on the State 3 V_O_ of isolated yeast mitochondria is rather insignificant^39^. In the same work, 0.3 mM ADP still allowed the observation of State 4 – State 3 – State 4 transitions (although State 3 was prolonged and its V_O_ increased), as ATP at concentration below 0.5 mM constituted a weak inducer of H^+^ permeability. Interestingly, ATP (2 mM) can also be involved in the activation of some cation (essentially K^+^ and H^+^)-conducting pathways present in the IMM of yeast but from the matrix side, causing an uncoupling effect regardless of OXPHOS inhibition^59^^,^^61^^,^^63^. However, such a scenario is rather unsuited to the OXPHOS measurements originally presented here, i.e. with high phosphate concentrations (10 mM) and increasing ADP levels (up to 1 mM) in the medium. This is because the ATP-dependent uncoupling effect (elicited from the matrix side) reported earlier is possibly completely prevented by both ADP (IC_50_ = 0.25–1 mM) and phosphate (PO_4_^3-^ from 4 mM was fully inhibitory, and up to 10 mM was used)^59^^,^^61^^,^^63^. Energy dissipation *via* some carriers/channels in yeast mitochondria caused by purine nucleotides (considering possible effects of GDP, GTP and ATP) exerting their effects from the intermembrane space was also alleviated/blocked/reversed by phosphate (up to 10 mM was used to obtain total inhibition) and ADP (up to 4 mM was tested)^39^^,^^40^^,^^58–60^^,^^62^. However, for yeast mitochondria, ATP (0.4–2 mM) has also been recognised as a nonuncoupling factor (in the presence of OXPHOS inhibitors), where ATP addition results in a stable ΔΨ or builds it up^40^^,^^42^^,^^63^. Moreover, as mentioned previously, yeast cytochrome *c* oxidase is also hampered by increasing ATP levels^44^. Curiously, in the research system employed in this work, the absence of ATP (0.35 or 2 mM) resulted in an unstable and decreasing V_O_ and ΔΨ of State 4, indicating time-dependent gradual inhibition of the respiratory chain without this purine nucleotide. Indeed, in isolated yeast mitochondria, ATP (1–2 mM) may be necessary to obtain a steady ΔΨ and augment it (in the presence of OXPHOS inhibitors), which can be coupled with an increase in V_O_^40^^,^^63^_._ Accordingly, the impact of ATP on mitochondria (acting from both sides of the IMM) is complicated, and the apparent changes in V_O_ and ΔΨ during bioenergetic studies have a pleiotropic background depending on the measurement conditions and yeast strain. The concentrations of purine nucleotides and phosphates used may be a key consideration and a reason for discrepancies among results derived from various research groups. Furthermore, GTP did not act as a stimulating or uncoupling factor in the currently described studies (data not shown) on isolated yeast mitochondria, although these features were previously attributed to GTP^40^^,^^58–60^. Considering the original results presented in this work, which were obtained in a medium favourable for OXPHOS, and those reported by other authors under OXPHOS exclusion^58^^,^^59^, GDP is mainly a stimulating molecule in intact yeast mitochondria. In other words, in properly functioning mitochondria, for instance, NDPK is typically targeted by GDP compared to AAC.

Interestingly, independent and variously designed studies revealed practically no effects of GDP^5–8^^,^^10^^,^^43^^,^^45–47^^,^^64^ or a physiologically irrelevant action of this purine nucleotide^1^^,^^9^^,^^51^^,^^65^ on AAC-perpetrated futile H^+^ uptake. According to both older and the newest data, ATP and ADP, the two highly transportable molecules for AAC, can be much more effective native and negative regulators of AAC uncoupling activity (on the basis of different approaches)^5^^,^^6^^,^^9^^,^^12^^,^^13^.

Considering the possibility of very rapid GDP metabolism in mitochondria, both at high (1 mM) and low (approximately 50–200 µM) GDP concentrations, described here and in earlier reports concerning various organisms (uni- and multicellular)^1^^,^^51–54^^,^^65^, the findings mentioned above that GDP is probably not a physiological and robust inhibitor of AAC-mediated H^+^ leak have been strongly supported from another point of view in this paper. Therefore, the observed promiscuity between GDP/CATR and their classical protein targets capable of uncoupling, i.e. the AAC/UCP (including the lack of GDP inhibitory effect after CATR and thus no additivity of GDP and CATR actions), needs further investigation to explain how GDP in fact (directly or indirectly) negatively affects AAC uncoupling activity^7^^,^^9–13^^,^^46^^,^^48^^,^^49^^,^^51^.

Finally, these findings fill the NDPK substrate specificity gap identified in 1991, where GDP was revealed as a suboptimal substrate for the purified NDPK of *S. cerevisiae*^23^. However, these authors highlighted that the subcellular compartmentation of NDPK can be a crucial determinant of its biological function, which is supported by the research presented here. In isolated mitochondria from wild-type yeast, GDP (0.2–1 mM) is a relevant substrate for NDPK (Figures 3A and 4A,B,D).

### OXPHOS inhibitors suppress mtNDPK activity and remodel GDP function

It was originally established in mammalian mitochondria many years ago, that the known inhibitors of AAC-mediated purine nucleotide transport, such as CATR and P-CoA, also inhibited AAC-sustained H^+^ leak induced by exogenous FFAs, however in the presence of another inhibitor of OXPHOS, i.e. oligomycin^5^^,^^6^. Moreover, so-called “basal” H^+^ leak mediated by AAC (without exogenous activators) and AAC-perpetrated H^+^ leak stimulated by the administration of FFAs as well as their peroxidation products, such as hydroxynonenal, are quenched by CATR molecules at least partly^4^^,^^7–13^^,^^45–47^. However, CATR is also able to inhibit a larger fraction of the given H^+^ leak or practically hamper it totally. In the present study, in mitochondria isolated from cells of the wild-type yeast strain, in the absence of oligomycin and exogenous FFA/hydoxynonenal, both CATR and P-CoA similarly impeded the capacity of endogenous mitochondrial H^+^ leak (generally accepted to be primarily sustained by AAC in yeast^4^) and therefore State 4 respiration, which drives H^+^ leak. Specifically, CATR (3.6 µM) decreased the V_O_ of State 4 by 37.5% and increased the ΔΨ of State 4 by 103%, and analogously, P-CoA (100 µM) decreased the V_O_ by 44.5% and increased the ΔΨ by 102%. Notably, to the best of my knowledge, the P-CoA inhibitory effect on innate H^+^ leak is presented here for the first time in yeast isolated mitochondria. However, these inhibitors simultaneously prevented (or reversed) the stimulatory effect of GDP, regardless of the purine nucleotide concentration (Table 2). Similarly, oligomycin (1–2 µg) also changed the parameters of State 4 respiration, i.e. it decreased V_O_ but only by 20.8%, increased ΔΨ by 101.9%, and finally counteracted the GDP stimulatory effect. Correspondingly, in isolated mammalian mitochondria, inhibitors of OXPHOS, such as CATR and oligomycin, likewise restrained the GDP stimulatory effect at different concentrations of this purine nucleotide^51^. Therefore, any lack of the GDP-provoked increase in V_O,_ accompanied by a decrease in ΔΨ in the presence of a given OXPHOS inhibitor, clearly demonstrates the relationship of the GDP-caused changes in mitochondrial respiration with OXPHOS.

Intriguingly, not only the inhibition of the OXPHOS system could be responsible for the abrogation of the GDP stimulatory effect *via* mtNDPK^57^. Almost sixty years ago, mtNDPK was reported to be sensitive to atractyloside (decarboxylated CATR) through the potential binding of this glycoside^66^. Nonetheless, during oxygraphic studies with isolated mitochondria, the direct interaction of CATR with mtNDPK may be imponderable because of the potential overlap of AAC and mtNDPK inhibition by this toxin^57^. The same could be true for conditions with P-CoA, as this compound directly inhibits two major AAC activities (independent transport of ADP/ATP and H^+^)^5^^,^^6^ as well as mtNDPK phosphotransfer activity^29^. In summary, among OXPHOS inhibitors, we can distinguish indirect blockers (targeting the OXPHOS apparatus) of the GDP stimulatory effect coupled with mtNDPK activity; however, potentially direct inhibitors of this kinase, such as CATR and P-CoA, cannot be excluded. Specifically, the adverse effects of CATR and P-CoA on OXPHOS, thus nucleotide metabolism, could be enhanced by the inhibition of the ADP generator, i.e. mtNDPK, in addition to the inhibition of AAC antiport activity.

Importantly, especially for measurements with CATR and P-CoA, the immobilisation of OXPHOS favoured a change in the GDP outcome; i.e. under such conditions, 1 mM GDP started to inhibit mitochondrial H^+^ leak (Table 2), as described previously in many articles, summarised in extensive review paper^1^. The inhibition of State 4 respiration supplemented with CATR or P-CoA by 1 mM GDP was comparable solely at the V_O_ level, i.e. in the presence of CATR, 1 mM GDP decreased V_O_ by 13.3% and increased ΔΨ by 101.2%, and in the presence of P-CoA, 1 mM GDP decreased V_O_ by 12.4% and very slightly but repetitively increased ΔΨ, i.e. by 100.2%; however, it is enough to ascertain that GDP in such a context is a recoupling factor. These findings also indicate that AAC uncoupling activity is not entirely stopped by CATR/P-CoA, as GDP additionally increased the inhibition of AAC-mediated H^+^ leak. Therefore, these findings provide further evidence that AAC is capable of GDP binding, i.e. nucleotide, which is probably not as specific as GTP is for UCP inhibition^51^. However, according to some results, both GDP and GTP can inhibit AAC-sustained H^+^ leak^9^. In summary, if mtNDPK does not rapidly metabolise GDP, it can have a physiological inhibitory effect on AAC-perpetrated H^+^ leak. Such a scenario predominantly occurs when OXPHOS somehow fails. Thus, GDP participates in the control of H^+^ electrochemical gradient consumption in mitochondria to promote energy conservation and limit energy dissipation (Figure 1, GDP-dependent rapid and late responses).

### Yeast mutants without NDPK

The lack of probably sole typical gene for NDPK in yeast cells is not a hindrance to success in mutant culture, regardless of the species, and mitochondria isolation^19^,^20^,^67^ (Sections “Yeast strains and culture conditions”, “Preparation of yeast spheroplasts” and “Isolation of yeast mitochondria”), as other kinases and NDPK-like proteins identified in different organisms could sustain essential life processes when classic NDPK is missing^16^^,^^19^^,^^67–72^. Therefore, considering two facts, i.e. encoding typical NDPK in baker’s yeast by only one gene^19^^,^^20^, and the relationship of the GDP stimulatory effect in isolated mitochondria with NDPK^51–54^^,^^65^, the exclusion of NDPK expression in such an organism should result in elimination of GDP-induced (indirectly *via* mtNDPK) OXPHOS. Indeed, the detailed findings presented in this paper revealed that in the absence of NDPK in yeast mitochondria, there was no GDP stimulatory effect regardless of the GDP concentration (Figures 3B and 4C,D and Tables 1 and 2). However, an ADP (up to 1 mM) stimulatory effect (sensitive to OXPHOS inhibitors) was observed in mitochondria isolated from the yeast mutant (Figures 3B,D and 4C and Table 1), where mutant State 3 respiration with 1 mM ADP was captured at V_O_ = 228.3 ± 40.3 (less than half that of mitochondria isolated from cells of the wild-type yeast strain, where V_O_ = 500.3 ± 28.1) and ΔΨ = 155.1 ± 5.3, which was more similar to the related parameter of the wild-type isolated mitochondria, i.e. ΔΨ = 161.8 ± 2.9 (Table 1). Concomitantly, the initial and stabilised State 4 respiration of mutant isolated mitochondria (with a saturated ATP concentration, i.e. 2 mM) was clearly lower, especially V_O_, than that of the analogous State 4 respiration of congenic wild-type yeast isolated mitochondria, i.e. the V_O_ reached 156.8 ± 8.8 (a decrease of 19.6%), and the ΔΨ reached 182.7 ± 2.8 (a decrease of 5.8%), which could mirror some of the general influence of genetic manipulation on mitochondrial physiology (Figures 3D and 4C,D and Table 1). Similar observations concerning V_O_ and ΔΨ changes, but measured separately and using other techniques for ΔΨ recording and in cells (not in isolated mitochondria), were reported for some human cancer models in the context of NDPK-D deficiency^28^. However, the mutant mitochondrial coupling parameters obtained with 0.1 mM ADP, i.e. RCR = 3.3 ± 0.7 and ADP/O = 1.1 ± 0.05, were almost identical to those of the wild-type cell counterparts, where RCR = 3.2 ± 0.1 and ADP/O = 1.1 ± 0.05 (Table 1).

Interestingly, in reference to Section “OXPHOS inhibitors suppress mtNDPK activity and remodel GDP function”, the oxygraphic measurements of mitochondria isolated from yeast mutants lacking NDPK, which did not reveal the GDP stimulatory effect, can somehow mirror the conditions with OXPHOS inhibitors established for the isolated mitochondria of wild-type yeast. Specifically, when 1 mM GDP cannot be rapidly metabolised *via* yeast mtNDPK, it starts to recouple for basal State 4 respiration (+ 2 mM ATP) and thus inhibits endogenous H^+^ leak (mainly AAC-sustained in yeast^4^) (Figure 4C,D and Tables 1 and 2). Specifically, 1 mM GDP, which is usually used to impede H^+^ leak, caused a decrease in V_O_, from 156.8 ± 8.8 to 139.2 ± 14.2 (by 11.2%), and the very small but repetitive increase in ΔΨ, from 182.7 ± 2.8 to 183 ± 3.5 (by 100.2%) compared with the initial State 4 respiration; thus, this inhibitory effect resembled the 1 mM GDP effect after P-CoA addition in isolated mitochondria of cells derived from the wild-type strain (Section “OXPHOS inhibitors suppress mtNDPK activity and remodel GDP function”). Therefore, continuing the conclusion from Section “OXPHOS inhibitors suppress mtNDPK activity and remodel GDP function”, any obstacles to rapid GDP metabolism may lead to the emergence of the additional bioenergetic function of GDP in the molecular physiology of cells, i.e. the inhibition of protein-mediated H^+^ leak. Specifically, such hindrance in rapid GDP metabolism could be (i) indirect effects caused, e.g. by various OXPHOS inhibitors affecting chemical reactions catalysed by mtNDPK (the strongly limited outflow of ADP when OXPHOS is stopped), (ii) direct inhibition of mtNDPK, or (iii) any mutation affecting mtNDPK expression, such as in the Δ*ynk1* model strain. Previous reports concerning the GDP inhibitory effect on H^+^ leak, both AAC- and UCP-catalysed, registered under conditions excluding OXPHOS^1^^,^^10–12^, support this idea. However, to capture the double-faced action of GDP, it is necessary to compare the effects of this nucleotide under different conditions, promoting or constraining OXPHOS and promoting or constraining GDP metabolism (mainly *via* NDPK), which are scenarios naturally encountered in living cells. This work is the first attempt to elucidate the complex functions of mitochondria in this context.

## Discussion

The key element of the presented studies is the nonclassic physiological-like medium used for H^+^ leak measurements, i.e. initially without OXPHOS inhibitors and supplemented with up to 2 mM exogenous ATP, in contrast to the most often used “classic” medium supplemented with oligomycin, which inhibits OXPHOS^1^^,^^51^. Owing to this modification of the conditions used to monitor the H^+^ leak phenomenon resulting from the respiration of isolated yeast mitochondria, not only the ADP but also the GDP metabolism involving OXPHOS was elegantly revealed. Principally, the GDP-induced changes in V_O_ and ΔΨ (convergent with the well-known ADP effect, i.e. increase in V_O_ and decrease in ΔΨ) described here for yeast could be evolutionarily well conserved, as they also concern amoebae, plants, and mammals^1^^,^^51^. The chosen method involves approaching physiological conditions without the influence of exogenous toxins such as CATR and oligomycin. The pleiotropic role of mtNDPK is the most substantial part of the paper, and different methodologies, i.e. the selective usage of nucleotides (ADP, ATP, GDP, and GTP), OXPHOS inhibitors (CATR, oligomycin, and P-CoA), and the proper mutant yeast strain, supported the idea that the OXPHOS apparatus and mtNDPK are involved in the GDP-dependent stimulatory effect in mitochondria. As AAC-mediated nucleotide exchange negatively regulates AAC-perpetrated H^+^ leak^8^^,^^9^, mtNDPK, which may channel ADP to AAC, helps maintain the “tightness” of the H^+^ electrochemical gradient and counteracts energy dissipation. Simply, the GDP-induced activity of mtNDPK promotes OXPHOS and synchronously decreases the magnitude of H^+^ leak in mitochondria, as AAC is principally implicated in ADP/ATP turnover. Moreover, in organisms that possess UCP, the high activity of mtNDPK could provide many GTP molecules, which are potent inhibitors of these typical carriers involved in futile mitochondrial H^+^ uptake^36^^,^^51^^,^^73^. In addition, OXPHOS, which is in progress, not only maintains the energy-demanding processes of the cell but also lowers the electrochemical H^+^ gradient across the IMM, which substantially limits the uncoupling activity of both AAC and UCP. Accordingly, fine-tuned cooperation of mtNDPK and AAC, possibly affecting the UCP if it is present in mitochondria, could be crucial to survival. The data presented here are of a physiological dimension, as different protein-originated effects are not separated. Owing to the mechanistic exclusion (caused by the addition of OXPHOS inhibitors) of essential mitochondrial proteins, which are key for obtaining a holistic understanding of the importance of H^+^ leak, including its inhibition by GDP, the immensely significant influence of mtNDPK on this phenomenon was not observed until 2014^51^.

Interestingly, although the bifunctional and opposite nature of GDP is observed in mitochondria, i.e. GDP, depending on conditions, is stimulatory or inhibitory for respiration (considering that H^+^ leak results from respiration), this nucleotide apparently promotes the energy conservation pathway. Specifically, GDP, as the secondary substrate (the first is most often ATP) for mtNDPK, which binds after ATP dephosphorylation and ADP release, indirectly (as, in fact, ADP) induces OXPHOS. Conversely, unmetabolized GDP directly counteracts H^+^ leak by affecting AAC/UCP-mediated uncoupling to support OXPHOS efficiency under conditions that limit OXPHOS (Figure 1). Importantly, if GDP inhibits H^+^ leak in yeast lacking UCP, it allows us to draw a general conclusion that GDP can negatively, *via* direct binding, control both major catalysts of futile H^+^ uptake, AAC and UCP, and thus GDP is specific for both carriers as considered earlier^1^^,^^10–12^. Results presented here support the assumption that GDP could be a critical physiological inhibitor of AAC-mediated H^+^ leak. In particular, this guanine nucleotide, under exceptional circumstances, such as mtNDPK/nucleotide metabolism dysfunction, including OXPHOS disturbances, e.g. mild CATR intoxication, may create a “GDP-dependent rapid response” to immediately counteract AAC/UCP-mediated H^+^ leak (to cause a decrease in mild uncoupling), which will be followed by a “GDP-dependent late response”, i.e. stabilisation of energy status, allowing it to maintain the balance between energy conservation to survive and energy dissipation to avoid excessive release of ROS (Figure 1).

Finally, our understanding of the previously poorly recognised effect of GDP on mitochondrial physiology has increased. The importance of nucleotide metabolism sustained by mtNDPK in the context of mitochondrial H^+^ leak may be crucial for increasing the commercial potential of yeast, similar to microalgae^74^, or for better understanding human health, including anticancer strategies and antiobesity medications^9^^,^^14^^,^^15^^,^^18^^,^^29–32^. Disturbances in mitochondria, including mutations in mitochondrial DNA, lead to the deregulation of cellular energy transduction processes and are characteristic factors of cancer that increase its progression^75^. Notably, the typical mitochondrially localised NDPK of mammals (NME4 or NDPK-D) is a recently discovered, novel metastasis suppressor in mammalian cells, the first attributed to mitochondria^28^, and NME6, also reported to be located inside mitochondria, may have similar functions^32^. Principally, the proper channelling of GTP to dynamins, owing to NDPK-D, is seen as antimetastatic, as it may counteract mitochondrial network fragmentation merged with cancer progression^30^^,^^31^. Therefore, exploring the nature of NDPK may shed new light on the potential development of novel therapeutics against different tumours. Moreover, the studies presented here concerning a model organism for eukaryotes, i.e. yeast, simulate changes in the molecular physiology of mitochondria, particularly alterations in nucleotide metabolism, affected by certain exogenous toxins, e.g. CATR^57^, or *de novo* and inherited mutations of mtNDPK.

## Conclusions

Model eukaryotic systems, such as the wild-type and mutant with a deleted gene for the only typical NDPK, have never been tested before in the context of the influence of NDPK on bioenergetics, i.e. changes in V_O_ and ΔΨ monitored simultaneously in isolated mitochondria under conditions favouring or excluding OXPHOS. This challenge constituted the central purpose of the research. The most important result of this study is the clear relationship between the GDP stimulatory effect and the presence of NDPK in mitochondria, where this vital enzyme cooperates with the OXPHOS apparatus and affects the H^+^ leak. The following main highlights can be drawn from this work:

- NDPK is key for GDP-induced, but ADP-mediated, OXPHOS in mitochondria;
- NDPKs, AACs, and OXPHOS apparatuses are crosstalk partners;
- the absence of NDPK in mitochondria may only slightly affect energy transduction;
- a lack of GDP metabolism may cause GDP to be inhibitory for mitochondrial H^+^ leak;
- OXPHOS inhibitors indirectly (directly?) hamper NDPK activity.

## Data Availability

The main text contains all the necessary data to evaluate the results. Additional data that support the findings are available from the corresponding author upon reasonable request.
